# Comparison of anterior cervical diskectomy with fusion (ACDF) and laminoplasty treating multilevel cervical spondylotic myelopathy with developmental canal stenosis: a retrospective study

**DOI:** 10.1186/s13018-023-04510-0

**Published:** 2024-01-03

**Authors:** Liping Dai, Chao Qin, Peiyu Guo, Hongda Gong, Weizhou Wang, Xiaodong Hou, Kaili Du, Chunqiang Zhang

**Affiliations:** 1 Department of orthopaedics, First Affiliated Hospital of Kunming Medical University, NO.295 Xichang Road, Kunming, 650032 Yunnan China; 2 Department of orthopaedics, Fujian Medical University Union Hospital, Antai Street, Fuzhou, 350001 Fujian China

**Keywords:** Multilevel cervical spondylotic myelopathy 1, Developmental canal stenosis 2, Anterior cervical diskectomy with fusion 3, Laminoplasty 4, Reserving space for the spinal cord 5

## Abstract

**Purpose:**

To evaluate clinical effectiveness and radiologic results of anterior cervical diskectomy with fusion (ACDF) comparing with laminoplasty (LP) in treating multilevel cervical spondylotic myelopathy (MCSM) with developmental canal stenosis (DCS).

**Methods:**

This was a retrospective analysis of 41 patients who had MCSM with DCS treated with ACDF or LP from December 2018 to April 2023. Patients were split into ACDF and LP groups for comparison, and patients were further separated into subgroups based on whether or not a reserving canal space was present. The operation time, hemoglobin, hospital stay, modified Japanese Orthopaedic Association (mJOA) score, and visual analog scale (VAS) score were used to assess clinical efficacy. The C2–C7 Cobb angle, C2–C7 sagittal vertical axis, T1 slope, and cervical range of motion were applied to evaluate imaging changes.

**Results:**

Of the 41 patients, 19 received ACDF, and 22 received LP. At the final follow-up, both groups’ mJOA scores significantly improved, and the intercomparison showed no differences; the VAS score was much lower in the ACDF group but remained unchanged in the LP group. At the final follow-up, the C2–C7 Cobb angle and T1 slope had significantly increased in the ACDF group, while the LP group showed no change; the cervical range of motion had significantly decreased in both groups, with the ACDF group exhibiting a more marked reduction. Within the ACDF subgroup, there was no postoperative symptom improvement for those with reserving space, whereas there was postoperative symptom resolution for those with non-reserving space; however, postoperative symptom in the LP subgroup was resolved.

**Conclusions:**

Both ACDF and LP were efficacious for MCSM patients with DCS. While ACDF could improve cervical lordosis and alleviate neck pain more effectively, it can also result in cervical sagittal imbalance and decreased mobility. Furthermore, the recovery from LP was superior to that from ACDF for patients with reserving space. In contrast, the recovery from both decompression techniques was comparable for individuals in non-reserving space.

## Introduction

Degenerative structures in the cervical spine compress the spinal cord or the supplying blood vessels, resulting in various symptoms, including sensory, motor, reflex, even bowel, and urine problems. This condition is known as cervical spondylotic myelopathy (CSM). Multilevel cervical spondylotic myelopathy (MCSM) is caused by cervical degenerative processes compressing several spinal cord segments. A significant contributing element to the development of spinal cord cervical spondylosis is developmental canal stenosis (DCS). The diagnostic standard for cervical spinal stenosis, the Pavlov ratio, is less than 0.75 [1]. Surgical options for patients of MCSM with DCS include anterior, posterior, or combination surgery, contingent on the patient's disease condition [2]. Both anterior diskectomy with fusion (ACDF) and posterior laminoplasty (LP) are crucial surgical treatments for MCSM with DCS, yet there is currently debate regarding which is better. Therefore, this study aims to evaluate the benefits and drawbacks of ACDF and LP in patients of MCSM with DCS.

## Results

This study enrolled 41 individuals of MCSM with DCS (ACDF group: 19 individuals and LP group: 21 individuals). There were no statistical differences in gender, average age, and C3–C6 Pavlov ratio between the ACDF and LP groups (*P* > 0.05). The ACDF group’s operating time was longer than the LP group's (*P* = 0.008). The two groups were similar regarding the HB reduction. Hospital stay was more extended in the LP group (*P* < 0.001) (Table 1).

**Table 1:** Comparison of basic indexes between the ACDF and LP groups

Comparative indicators	ACDF	LP	*P*
Gender (male/female)	6/13	13/9	0.078
Age (years) *X* ± S.D	57.37 ± 8.53	57.41 ± 9.29	0.988
Pavlov ratio P50 (P25, P75)	0.65 (0.59, 0.69)	0.64 (0.59, 0.69)	0.675
Operation time (hours)	3.50 (3.00, 5.00)	2.00 (2.00, 3.63)	0.008^†^
HB reduction level (g/L)	21.05 ± 12.06	20.73 ± 12.40	0.933
Hospital stay (days)	6.00 ± 2.47	10.5 ± 4.95	< 0.001^†^

ACDF, Anterior cervical diskectomy with fusion; LP, Laminoplasty; HB, Hemoglobin; *P*, *P* value; P50, Median; P25, 25th percentile; P75, 75th percentile; and *X* ± S.D., Mean ± standard deviation

“^†^” means *P* < 0.05, which is statistically significant

### Clinical outcomes

The ACDF and LP groups had comparable preoperative mJOA and VAS scores. The two groups’ mJOA scores increased dramatically compared to the preoperative period, and there was no difference in scores between them after surgery. VAS scores in the ACDF group considerably declined after surgery (*P* = 0.012), while the LP group did not significantly alter before and after the procedure (*P* = 0.079). The recovery rate was similar in both groups (Table 2).

**Table 2:** Comparison of clinical indexes between the ACDF and LP groups

Comparative indicators	ACDF	LP	*P*
mJOA score			
Preoperative	12.00 (11.00, 13.00)	12.25 (10.75, 13.00)	0.078
Final follow-up	14.50 (11.00, 15.00)	14.00 (12.00, 15.00)	0.958
*P*	< 0.001^†^	< 0.001^†^	
VAS score			
Preoperative	3.00 (0.00, 5.00)	0.00 (0.00, 6.00)	0.297
Final follow-up	1.00 (0.00, 3.00)	0.00 (0.00, 2.00)	0.611
*P*	0.012^†^	0.079	
Recovery rate (%)	39.18 ± 38.03	39.83 ± 45.66	0.961

“^†^” means *P* < 0.05, which is statistically significant

ACDF, Anterior cervical diskectomy with fusion; LP, Laminoplasty; mJOA, Modified Japanese Orthopaedic Association; VAS, Visual analog scale; and *P*, *P* value

### Imaging indexes

The preoperative C2–C7 Cobb angle, SVA, T1 slope, and cROM (cervical range of motion) of the ACDF and LP groups were identical. When comparing the final follow-up to the preoperative period, the ACDF group’s C2–C7 Cobb angle and T1 slope were considerably higher (*P* = 0.039 and* P* = 0.026), while the LP group showed no change. At the final follow-up, there was no difference in SVA between the two groups from the preoperative data. The cROM dropped in both groups when comparing the preoperative period to the final follow-up (*P* < 0.001). However, the ACDF group's drop was more noticeable than the LP group’s (Table 3).

**Table 3:** Comparison of imaging indexes between the ACDF and LP groups

Comparative indicators	ACDF	LP	*P*
C2–C7 Cobb angle (°)			
Preoperative	15.95 ± 8.51	14.86 ± 8.60	0.688
Final follow-up	21.85 ± 9.94	15.14 ± 8.65	0.025^†^
*P*	0.038^†^	0.778	
SVA (mm)			
Preoperative	14.51 ± 8.47	18.31 ± 9.65	0.191
Final follow-up	18.94 ± 12.56	22.34 ± 15.00	0.443
*P*	0.118	0.548	
T1 slope (°)			
Preoperative	23.00 ± 7 .23	25.95 ± 7.54	0.210
Final follow-up	27.00 ± 5.93	23.86 ± 7.39	0.146
*P*	0.026^†^	0.093	
cROM (°)			
Preoperative	43.16 ± 8.86	45.55 ± 10.53	0.380
Final follow-up	18.47 ± 6.10	40.55 ± 8.87	< 0.001^†^
*P*	< 0.001^†^	< 0.001^†^	

ACDF, Anterior cervical diskectomy with fusion; LP, Laminoplasty; SVA, C2–C7 sagittal vertical axis; cROM, Cervical range of motion; and *P*, *P* value

“^†^” means *P* < 0.05, which is statistically significant

### ACDF subgroup

Within the ACDF group, five individuals were in the space-reserving subgroup, and fourteen were in the non-space-reserving subgroup. The two subgroups' preoperative mJOA scores were comparable, while, at the final follow-up, the mJOA scores differed significantly (*P* = 0.007). The space-reserving subgroup showed no change in the final follow-up mJOA scores from preoperative (*P* = 0.736). In contrast, the non-space-reserving subgroup substantially increased in the final follow-up mJOA scores (*P* < 0.001). Additionally, there was a noteworthy distinction between the two subgroups for recovery rate (*P* = 0.000) (Table 4).

**Table 4:** Comparison of space-reserving and non-space-reserving subgroups in the ACDF group

	*N*	Recovery rate	Preoperative mJOA score	mJOA score at final follow-up	*P*
Space-reserving	5	− 6.88 ± 32.10	11.30 ± 1.82^†^	11.00 ± 2.62^†^	0.736^†^
Non-space-reserving	14	55.63 ± 24.04	11.82 ± 1.97^†^	14.50 ± 2.04^†^	< 0.001^†^
*P*		0.000	0.612	0.007	

“^†^” means the p-value corresponding to both groups' mJOA score at preoperative and final follow-up; *P*, *P* value and mJOA, Modified Japanese Orthopaedic Association

### LP subgroup

Twelve individuals in the space-reserving subgroup and ten in the non-space-reserving subgroup in the LP group had similar preoperative mJOA scores. At the final follow-up, there was a significant increase in scores in both subgroups (*P* < 0.05), with the non-space-reserving subgroup showing a more noticeable increase (*P* = 0.036). The recovery rates in the two subgroupings were comparable (*P* = 0.056) (Table 5).

**Table 5:** Comparison of space-reserving and non-space-reserving subgroups in the LP group

	*N*	Recovery rate	Preoperative mJOA score	mJOA score at final follow-up	*P*
Space-reserving	12	22.99 ± 50.55	11.29 ± 2.66^†^	12.96 ± 2.16^†^	0.038^†^
Non-space-reserving	10	60.05 ± 30.13	12.15 ± 1.83^†^	14.85 ± 1.70^†^	< 0.001^†^
*P*		0.056	0.398	0.036	

*P*, *P* value and mJOA, Modified Japanese Orthopaedic Association

“^†^” means the *P* value corresponding to both groups' mJOA score at preoperative and final follow-up

In addition, while the age at disease was similar for reserving and non-reserving space patients (*P* = 0.689), the non-reserving space patients' disease duration was much shorter (*P* = 0.040) (Table 6).

**Table 6:** Comparison of all patients on reserving and non-reserving space

	Disease duration (months)	Age at disease (years)
Reserving space	9.00 (5.50, 24.00)	58.06 ± 7.26
Non-reserving space	6.00 (2.00, 12.00)	56.92 ± 9.92
*P*	0.040	0.689

*P*, *P* value

## Methods

**Fig. 1:**
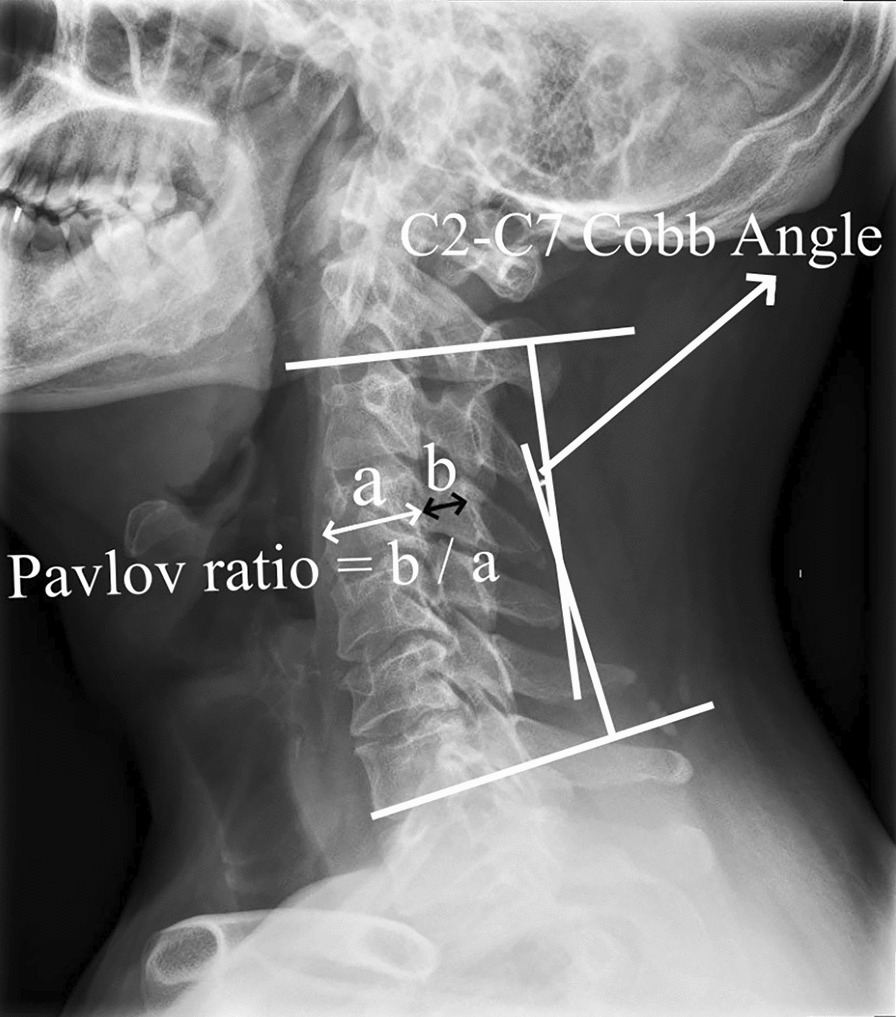
Preoperative radiographs of mCSM with DCS; the figure shows the measures of the Pavlov ratio and the C2–C7 Cobb angle (angle of intersection of C2 and the extension of the lower end plate of C7)

**Fig. 2:**
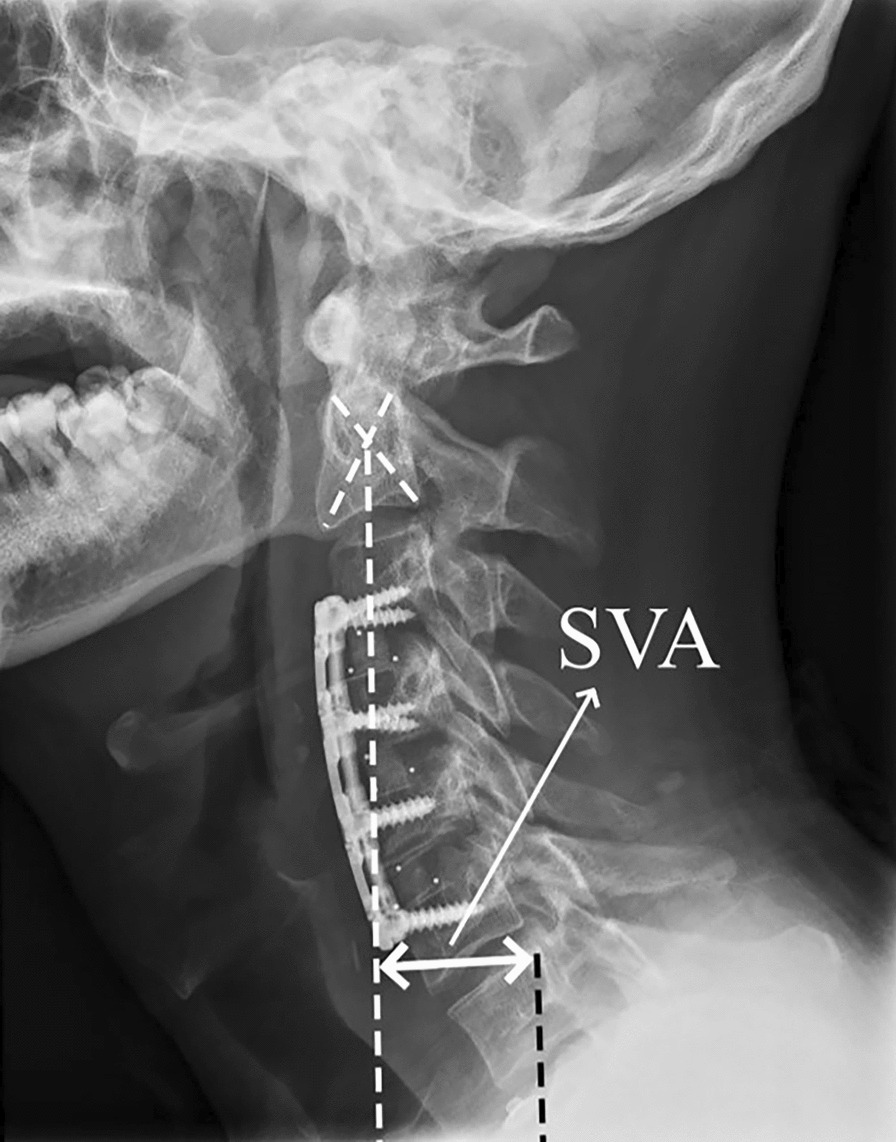
Postoperative three-segment ACDF X-rays also show SVA measurements (the distance between a plumb line dropped from C2 and the posterior–superior corner of C7)

**Fig. 3:**
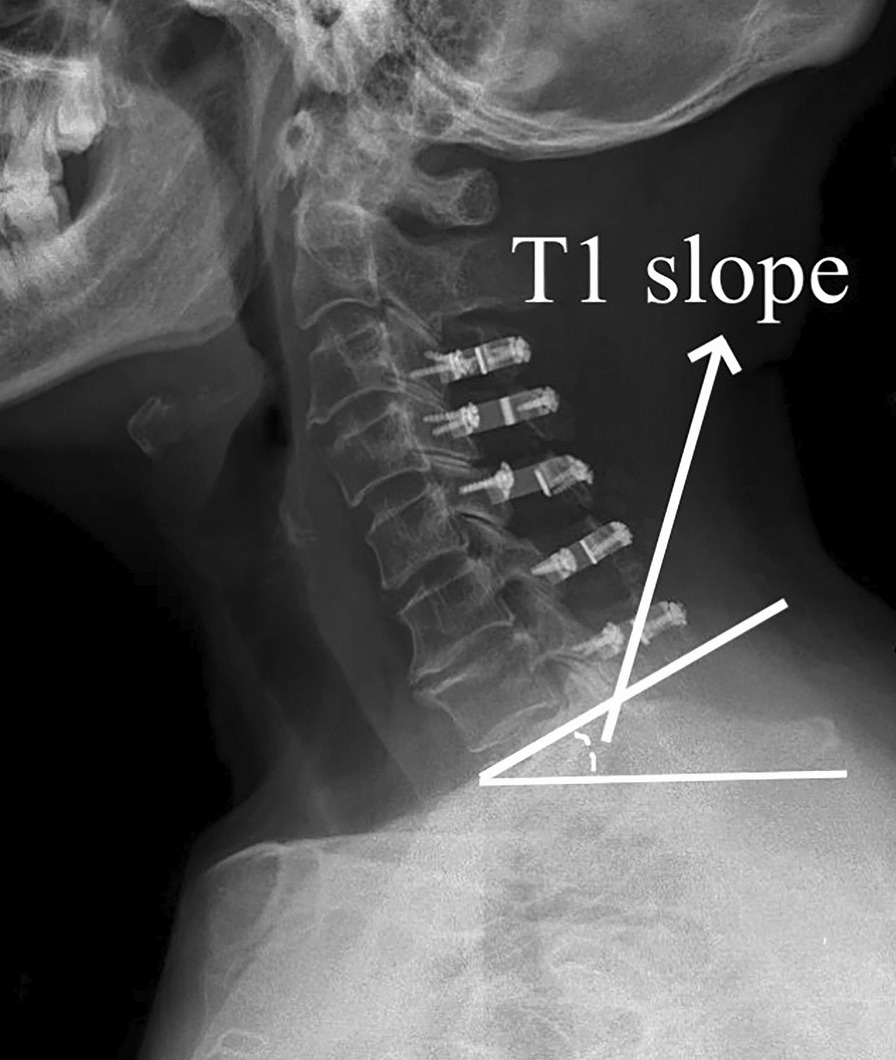
LP postoperative radiographs, which include measurements of the T1 slope (the angle between parallel end plate lines and horizontal reference lines on T1)

**Fig. 4:**
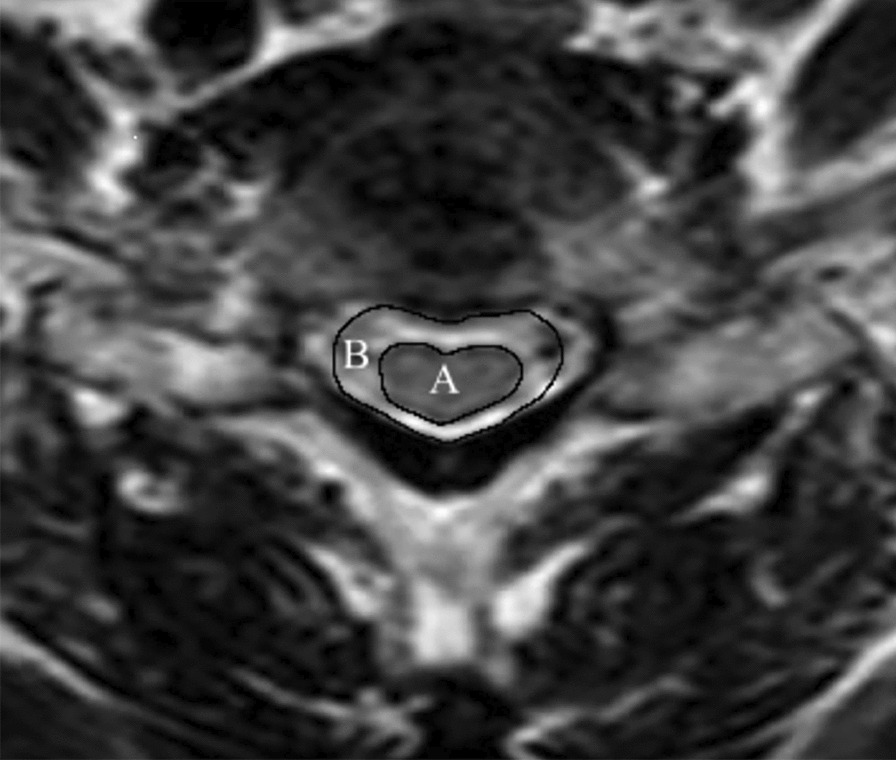
**A** The transverse area of the spinal cord and **B** the transverse area of the dural sac on T2-weighted axial MRI

In this study, we reviewed patients who received ACDF or LP in our department between December 2018 and April 2023 and had a diagnosis of MCSM with DCS (Fig. 1) with a mean follow-up of 24 months (range: 6 months–57 months). Every patient was followed. Depending on the type of surgery, they were split into two groups: the LP group (n = 22, Fig. 3) and the ACDF group (n = 19, Fig. 2). The situation was evaluated before surgery and after final follow-up using the modified Japanese Orthopaedic Association (mJOA) scale. Recovery rate (%) = [Postoperative score − Preoperative score]/[Perfect score (17) − Preoperative score] × 100 is the formula that was utilized to calculate the improvement rate of surgery. The visual analog scale (VAS) was used to evaluate neck pain. The C2–C7 Cobb angle (Fig. 1), C2–C7 sagittal vertical axis (SVA) (Fig. 2), and T1 slope (Fig. 3) were applied to evaluate sagittal balance [3] in upright lateral cervical radiographs. The modified cervical range of motion (cROM) method was used to measure the cervical range of motion. On the full supination and flexion upright lateral cervical radiographs, two straight lines were drawn along the lowest point of the anterior margin of the C2 and C7 vertebrae to the lowest point of the posterior inferior angle of their spinous processes, respectively. The angles at which the two straight lines intersected were the angles of supination and flexion (the angle of flexion: Anterior convexity is positive, and posterior convexity is negative) and cROM = supination − flex angle. In the same vein, preoperative radiographs’ C3–C6 Pavlov ratio was applied to determine the degree of DCS [4], Pavlov ratio = sagittal diameter of the cervical spinal canal/sagittal diameter of the cervical vertebral body (Fig. 1). Subgroups were then created for each group based on whether there was reserving space or not in spinal canal [5] [The spinal cord/dural sac area ratio on magnetic resonance imaging (MRI) film of patients with DCS was < 0.41, and ≥ 3 segments were considered as still having reserving space; otherwise, it was non-reserving space, and measurement ranged from C2 to C7 (Fig. 4)]. Then, the comparisons of clinical results were made between the subgroups. The difference in operation time, hemoglobin (HB) reduction level, and hospital stay (defined as the time from the surgery to discharge) were also employed as additional evaluation indicators. Each patient and family member willingly signed an informed permission form. All the data for this study were gathered by computing the mean value based on the statistics of three doctors in our team.

The inclusion criteria were as follows: 1. With a definitive diagnosis of MCSM (≥ 3 segments) accompanied with DCS; 2. Undertaking LP (more than 3 segments) or multi-segmental ACDF (≥ 3 segments); 3. Undergoing conservative treatment at least half a year without satisfying results, or the symptoms adversely impacting the patients; and 4. Integral imaging and clinical data were collected. The exclusion criteria were as follows: 1. A history of prior cervical spine surgery; 2. Simple radiculopathy, posterior longitudinal ligament or ligamentum flavum ossification, and cervical kyphosis; 3. Disk herniation or osteophyte with occupation rate > 50% [6]; 4. Combined with severe illnesses or ailments impacting the nervous system; and 5. With previous post-traumatic myelopathy or in conjunction with further cervical spine disorders.

### Surgical technique

After undergoing general anesthesia, each patient in the ACDF group had numerous segments (3–5 segments) operated on. The patient was supine, and a right anterior cervical transverse incision was performed to expose the vertebral body, and the intervertebral disks were removed. The intervertebral space was filled with an allograft-filled interbody fusion, and titanium plates and screws were used to hold the vertebral bodies in place (Fig. 2). In the LP group, 12 patients underwent C3–C7 LP, and 10 underwent C3–C6 or C4–C7 LP. The skin was incised through a posterior median incision in the prone position, then gradually separated to the vertebral plate. Decompression was performed using a single-opening laminoplasty. The vertebral plate was opened on the one side and hinged on the other. Titanium mini-plates were used to keep the spinal canal enlarged, and screws were used to secure the plates (Fig. 3). Routine placement of drains followed by closure of the incision. Two qualified surgeons in our team performed the surgeries for this study.

### Statistical analysis

Using SPSS 27, the analysis was carried out. The differences between the two gender groups were compared using the chi-square test, the differences between the two groups were compared using the independent samples *t*-test, and the differences between the groups’ pre- and post-surgery differences were compared using the paired samples *t*-test. Nonparametric tests were employed if the data did not fit the normal distribution: The Wilcoxon test was used for comparisons within groups, and the Mann–Whitney test was used for comparisons between the two groups.* P* < 0.05 was used to establish statistical significance.

## Discussion

ACDF and LP are equally effective treatments for MCSM [7]. Zhang et al. [8] reported that ACDF can achieve the same level of surgical success for MCSM patients with DCS as for patients without DCS; that is, DCS does not affect the improvement of neurological function following surgery. Shigematsu et al. [9] considered outcomes of double-door LP unaffected by DCS. In our study, the LP and ACDF groups had significantly higher mJOA scores and improvement rates.

This study also discovered that the LP group showed no sign of a significant reduction in neck pain at follow-up, which is consistent with the study of Liu et al. [10] and Woods et al. [11]. One of the main reasons lay in how much posterior neck muscles were damaged in the procedure. Numerous factors contribute to neck discomfort, which is frequently complex [12]. Therefore, more research is required to determine the causes of the neck pain that persists following LP. Maybe spinal imbalance is another reason.

Key markers of spinal balance include cervical lordosis and T1 slope. Spinal imbalance is indicated by a T1 slope of more than 25° or less than 13° [13]. According to Chen et al. [14], the C2–C7 Cobb angle and T1 slope have a positive correlation. Sakai et al. [15] demonstrated that a key risk factor for postoperative kyphosis is an imbalance in the cervical sagittal plane. In our study, the T1 slope considerably increased in the ACDF group (28.58°). As a result, multi-segmental ACDF may result in sagittal imbalance of the cervical spine, which is another issue to consider when choosing the anterior approach for MCSM with DCS. The visualization of T1 slope is influenced by body shape, posture, and radiography level. About thirty percent of patients do not exhibit the T1 slope clearly; this can be replaced by measuring C7 [16, 17].

Following LP, lordosis loss may transpire [18]. However, Liang et al. [19] felt that LP would not have an impact on cervical lordosis following surgery, and patients who had an Ishihara index (a radiographic evaluation tool for cervical lordosis curvature) less than 20 may have a bigger Cobb angle following ACDF. Shi et al. [20] also showed that following a four-segment ACDF, the Cobb angle rose. The results of our investigation also indicated that LP did not affect the cervical lordosis or the Cobb angle. ACDF can increase cervical lordosis. Consequently, we may think about performing multi-segmental ACDF to correct cervical lordosis in patients of MCSM with DCS who have poor cervical lordosis.

Lee et al. [21] proved that cervical mobility was more affected by three-segment ACDF than by LP. In our investigation, cervical mobility was impacted by both ACDF and LP; however, the impact of ACDF was greater than that of LP, which not only affected the patient's quality of life but also might have been a risk factor for the disease-developing other segments [21].

In a meta-analysis by Xu et al. [22], the operation time and blood loss after ACDF for MCSM were comparable to those from LP. We found that the ACDF may take longer operating time than LP, likely due to the narrower surgical field view, which requires longer times to address degenerating tissues. Our outcome is comparable to their in terms of bleeding. Because the LP was more traumatic and required more time for incision healing than the ACDF, it required a lengthier hospital stay in our study.

Reserving space is also a consideration for the choice of surgical procedure. Tang et al. [5] showed that recovery from anterior decompression surgery was better for individuals with preserving or normal intradural space. However, both anterior and posterior techniques improved clinical symptoms for individuals with non-reserving space. Similarly, Yu et al. [23] conducted a comparable investigation and reached the same conclusion. In our study, posterior surgery was more successful in patients with a reserving space, and anterior surgery was less effective. In contrast, anterior and posterior surgery were equally effective in patients with a non-reserving space. It is consistent with the *Spinal Surgery*, edited by Chen et al. [1]. In the study by Yu et al., ACDF was performed in one or two segments, and the choice of surgery was not specified for patients with DCS in the study by Tang et al. Therefore, this may have influenced the conclusions in a biased way. In our study, all of the patients underwent multi-segmental (≥ 3 segments) ACDF or LP after undergoing MCSM (≥ 3 segments) with DCS. Selection bias can be avoided more effectively this way. We believe that patients with a non-reserving space and shorter disease duration, the spinal cord can be directly or indirectly made more space by anterior or posterior decompression, improving patient recovery. Contrarily, individuals with reserving space, who have longer disease duration, degenerative hyperplastic tissue in their spinal canals is heavier; although anterior decompression directly decompresses the pressure-causing substance, it only releases a small amount of spinal cord space. After posterior decompression, there is a notable enlargement of the spinal canal [24] and a retrograde displacement in the spinal cord [25–27]. However, this also necessitates a longer period of follow-up to find out whether the patient will eventually experience any symptomatic recurrence.

## Limitation

Due to the small sample size and the fact that this was a retrospective study with short-term follow-up, the conclusions of this investigation may be limited. Clinical conclusions would have been stronger if a cohort study or a bigger sample size had been performed.

## Conclusion

Both ACDF and LP were efficacious for MCSM patients with DCS. While ACDF could improve cervical lordosis and alleviate neck pain more effectively, it can also result in cervical sagittal imbalance and decreased mobility. Therefore, ACDF is recommended to patients who have poor cervical lordosis or significant neck pain, and LP is recommended to people with good cervical lordosis. Furthermore, the recovery from LP was superior to that from ACDF for patients with reserving space. In contrast, the recovery from both decompression techniques was comparable for individuals in non-reserving space.

## Data Availability

The datasets used and/or analyzed during the current study are available from the corresponding author upon reasonable request.

## Abbreviations

ACDF: Anterior cervical diskectomy with fusion
LP: Laminoplasty
mJOA: Modified Japanese Orthopaedic Association
MCSM: Multilevel cervical spondylotic myelopathy
DCS: Developmental canal stenosis
cROM: Cervical range of motion
SVA: The C2–C7 sagittal vertical axis
VAS: The visual analog scale
MRI: Magnetic resonance imaging
SPSS: Statistical Package for the Social Sciences
HB: Hemoglobin
*P*: *P* value
